# High Prevalence of Multidrug-Tolerant Bacteria and Associated Antimicrobial Resistance Genes Isolated from Ornamental Fish and Their Carriage Water

**DOI:** 10.1371/journal.pone.0008388

**Published:** 2009-12-21

**Authors:** David W. Verner-Jeffreys, Timothy J. Welch, Tamar Schwarz, Michelle J. Pond, Martin J. Woodward, Sarah J. Haig, Georgina S. E. Rimmer, Edward Roberts, Victoria Morrison, Craig Baker-Austin

**Affiliations:** 1 Centre for Environment, Fisheries and Aquaculture Sciences, Weymouth Laboratory, Weymouth, Dorset, United Kingdom; 2 United States Department of Agriculture/Agricultural Research Service, National Center for Cool and Cold Water Aquaculture, Kearneysville, West Virginia, United States of America; 3 Division of Infection and Immunity, University of Glasgow, Glasgow, United Kingdom; 4 Veterinary Laboratories Agency, Addlestone, Surrey, United Kingdom; Charité-Universitätsmedizin Berlin, Germany

## Abstract

**Background:**

Antimicrobials are used to directly control bacterial infections in pet (ornamental) fish and are routinely added to the water these fish are shipped in to suppress the growth of potential pathogens during transport.

**Methodology/Principal Findings:**

To assess the potential effects of this sustained selection pressure, 127 *Aeromonas* spp. isolated from warm and cold water ornamental fish species were screened for tolerance to 34 antimicrobials. Representative isolates were also examined for the presence of 54 resistance genes by a combination of miniaturized microarray and conventional PCR. Forty-seven of 94 *Aeromonas* spp. isolates recovered from tropical ornamental fish and their carriage water were tolerant to ≥15 antibiotics, representing seven or more different classes of antimicrobial. The quinolone and fluoroquinolone resistance gene, *qnrS2*, was detected at high frequency (37% tested recent isolates were positive by PCR). Class 1 integrons, IncA/C broad host range plasmids and a range of other antibiotic resistance genes, including *floR*, *bla*_TEM−1_, *tet*(A), *tet*(D), *tet*(E), *qac*E2, *sul1*, and a number of different dihydrofolate reductase and aminoglycoside transferase coding genes were also detected in carriage water samples and bacterial isolates.

**Conclusions:**

These data suggest that ornamental fish and their carriage water act as a reservoir for both multi-resistant bacteria and resistance genes.

## Introduction

The trade in ornamental (pet) fish is greater then 1 billion animals per year globally [1]. More than 45 million fish per year are imported into the United Kingdom (UK) alone from a wide range of countries, in particular those in South East Asia. An estimated 14% of all UK households have an aquarium or, alternatively, a pond [2].

Antimicrobials are used by owners and retailers to directly control bacterial infections [3]. They are also routinely added to the water these fish are transported in to suppress the growth of potential pathogens during transport [4]. Trust and Whitby [5] noted that this practice was widespread more than thirty years ago. As a consequence, antibiotic tolerant bacteria have likely been selected for and proliferate in the trade [3], [5].

Currently, microbial resistance to antibiotics spans all known classes of natural and synthetic drug agents [6], and bacterial resistance to antibiotics continues to pose a serious threat to human and animal health [7]. Resistance can either arise from mutations in genes native to the chromosome of the bacterial species in which they are found, or by acquisition of transferable genetic elements (e.g. plasmids and/or resistance gene encoding integrons) [8]. It is known that either process can lead to the clonal expansion of resistant pathogens that affect humans and farmed animals, including fish.

Antibiotic resistance research has typically been very disease-focused, likely contributing to our limited understanding regarding the ecology, evolution and dissemination of antibiotic resistance in the environment [9]. However, studies have established that plasmids, integrons [10] and associated antimicrobial resistance (AMR) genes of bacteria recovered from the aquatic environment can share very high sequence homology to clinically important plasmids and AMR genes [11]–[17].

This suggests that there is resistance gene flow between aquatic and anthropogenic sources. The direction of this route of transfer is unknown, as are the potential health risks arising from this transfer.

Despite the increasing body of evidence regarding the role of transferable genetic elements in the dissemination of antibiotic resistance in pathogens that affect farmed fish, there is a relative paucity of data concerning their role in the development and transfer of resistance in pet fish. A recent Australian study also noted a possible a link between the ownership of ornamental fish and a limited number of multidrug resistant (MDR) *Salmonella* Java human infant cases [18].

It has been recommended that the risks associated with the transfer of antibiotic resistant bacteria through direct contact exposure to ornamental fish should be determined [19]. It is also important for the ornamental fish industry to recognize the extent to which the bacteria associated with ornamental fish have developed antimicrobial resistance.

Towards these overall aims, a characterization of antibiotic tolerance in *Aeromonas* spp. present in ornamental fish and carriage water samples was undertaken. *Aeromonas* spp. were selected as some species, e.g. *A. hydrophila*, include pathotypes of clinical significance for both fish and humans [20] and are also ubiquitous, representative members of the aquatic microbial community. As well as determining their tolerance to a range of antimicrobials, the presence of select resistance genes in isolates was determined using a miniaturized microarray. To give an indication of the extent to which the whole microbial communities present in carriage water may be enriched for genes conferring resistance to antimicrobials, a culture-independent characterization of class 1 integron diversity in water samples was also undertaken.

## Materials and Methods

### Samples

A total of 25 consignments, each containing different varieties and species of warm water ornamental fish, were sampled between February and April 2008. Fish species and associated carriage waters sampled included, guppies (*Poecilia reticulata* Peters), threadfin rainbow (*Iriatherina werneri* Meinken), celebes rainbow (*Telmatherina ladigesi* Ahl), neon gold barb (*Puntius semifasciolatus* Günther), harlequin rasbora (*Trigonostigma heteromorpha* Duncker), neon tetra species (*Paracheirodon innesi* Myers and *Hyphessobrycon herbertaxelrodi* Géry), red wag platy (*Xiphophorus maculates*), kuhli loach (*Pangio kuhlii* Valenciennes) and silver molly (*Poecilia shenops*).

These species are typically reared at temperatures between 24 and 30 °C. The fish were predominantly shipped to the UK from Singapore (19/25 samples), although wild caught species shipped from Columbia, Guyana and Brazil were also included. Bags were either intercepted at the UK's London Heathrow Airport en route to distributors, or the morning after they arrived in the UK from a local wholesale distributor (Weymouth, Dorset). Fish were kept and transported to the laboratory in their original carriage water in sealed bags in boxes, with enough oxygen to survive 24–48 h before sampling. Samples (10 µl) of carriage water and whole fish homogenised in phosphate buffered saline (PBS) were seeded onto solid *Aeromonas* media (Oxoid, Basingstoke UK). Resultant presumptive colonies were subcultured and confirmed as *Aeromonas* spp., based on phenotypic testing criteria (Gram negative, cytochrome oxidase and catalase positive, motile, rods able to ferment and oxidize glucose, with API 20NE system (Biomerieux, France) biochemical test profiles typical of *Aeromonas* spp.). A subset of 41 isolates, including all those described in Table 1, were further confirmed as *Aeromonas* spp., based on partial 16S rRNA gene sequencing [21]. The *gyrA* and *gyrB* genes of a further four of these isolates were partially sequenced, using previously described methods [22]. This was to confirm 16S rRNA gene based identifications and to identify potential mutations in the quinolone resistance-determining regions of these two genes. Apart from ampicillin, to which *A. hydrophila* is intrinsically resistant [23], no antibiotics were included in any of the isolation media used.

**Table 1:** Example bacterial isolates examined for presence of Class 1 integrons, Inc A/C plasmid markers and a range of resistance genes by miniaturised microarray and PCR.

Isolate No.	aIdentification	Origin	Country of origin	cResistance phenotype	Resistance genes, plasmid markers and class 1 integrons detected by miniaturized microarray and/or PCR
93022	*A. veronii*	Koi carp	UK	Amx, Otc, Tet, Oxa, Flq, Neo(I), Str (I), Spe, Stz, Tob(I), Fun	*bla_TEM1_ + tet*(E)*
93024	*A. hydrophila*	Koi carp	UK	Amx, Otc, Tet,Eno,Chl (I), Spe, Oxa,Flq,Tio, Sxt,Stz	*+ aadA1 + dfrA1 + intl1* * *+ sul1 + tet*(A)
94070	*A. hydrophila*	gold fish	UK	Amx,Otc, Tet(I),	*tet*(E)
98013	*A. veronii*	koi carp	UK	Amx,Otc, Tet, Cip,Eno,Gat (I), Flq,Oxa, Faz, Fox,Tio, Tans, Fun(I),	*bla_TEM1_* * *+ tet*(E)* *+ qnrS2* *
08015	*A. punctata*	guppy	Singapore	Amx,Eno, Cpr,Gat,Tio,Otc,Tet,Ffl,Fun, Ofl, Oxa,Flq,Faz,Fox,	*qnrS2* * *+ tet*(E)
08016	*A. hydrophila*	guppy	Singapore	Amx, Otc,Tet, Flq,Oxa,Eno,Cpr,Gat,Ofl, Fun, Tio(I),Neo(I),Chl,Ffl,Str,Tans,Gen, Tob(I),Spe,Stz,Sxt,	*dfr12 + intl1 +sul 1 + tet*(D) *+ tet*(E) *+ floR* **+** *qnrS2* *
08019	*A. punctata*	guppy	Singapore	Amx,Otc,Tet, Oxa, Flq, Cpr,Eno,Gat, Ofl, Fun,Pip(I),Tans,Tio,Chl,Ffl,Str(I),Stz,Sxt	*bla_TEM1_ + tet*(E)* *+ dfr12* * *+ floR* * **+** *int1*
08020	b *A. veronii*	guppy	Singapore	Amx,Otc, Tet, Flq,Oxa, Cip, Eno, Gat, Fun,Faz,(I),Tio(I), Tob(I)	*QnrS* * *+ tet*(D) *+ tet*(E) *+*IncA/C‡
08022	*A. hydrophila*	threadfin rainbow	Singapore	Amx,Otc,Tet, Oxa,Flq,Cpr,Eno,Gat,Ofl, Fun,Faz,Neo(I),Chl,Tio(I),Str,Stz,Sxt,	*dfr12* * *+ qnrS2* * *+ sul1 + bla_TEM1_ + tet*(E) *+ intl1* *
08030	b *A. hydrophila*	harlequin rasbora	Singapore	Amx,Otc,Tet(I), Oxa,Flq,Eno,Ofl(I), Flq,Oxa,Fox, Pip(I),Tio(I),Ami,Gen,Nov,Stz,Sxt	*dfrA1 + intl1 + orfC* † *+ arr2* †
08033	b *A. punctata*	redwag platy	Singapore	Amx,Otc,Tet, Flq,Oxa,Eno,Cpr,Gat,Ofl, Fun,Faz,Neo(I),Str,Chl,Gen,Stz,Sxt,	*VatE,+ ant21a + catB8 + strA,+ strB, tet*(G) *Intl1* * *+ qnrS2* * *+ bla_OXA7_ +sul1 + bla_TEM1_ + tet*(D) *+ dfr12 + aac6 lb + aadA1*
08038	*A. hydrophila*	three lined pencil	Guyana	Amx,Otc,Tet, Oxa,Flq,Cpr,Eno,Gat, Fun,Faz,Fox,Tio(I), Chl,Ffl(I),Neo(I),Ofl, Stz,Sxt	*dfr12 + Intl1* * *+ sul1 + qnrS2* * *+ tet*(D) * *+ tet*(E)
08039	*A. hydrophila*	silver hatchet	Guyana	Amx,Otc,Tet,Cpr,Eno,Gat,Ofl,Oxa,Flq,Faz,Fox,Tio,Ffn,Neo,Gen,Str,Stz,Sxt	*dfr12 + sul1 + qnrS2* * *+ tet*(E)
08041	*A. punctata*	Silver hatchet	Guyana	Amx,Otc,Tet, Flq,Oxa,Cpr,Eno,Faz,Fox,Gat,Gen,Ofl, Neo,Pod(I),Tio, Sxt	*bla_TEM1_ + tet*(E) *+tet*(A) *+ sul1 + aad A2* * *+ dfr12 + Intl1 + qnrS2* *
08043	b *A. hydrophila*	silver hatchet	Guyana	Amx,Otc,Tet,Eno,Cpr,Gat,Ofl,Oxa,Flq,Fun,Faz,Fox,Chl, Ffl(I), Str,Stz,Sxt	*QnrS2* * *+ tet*(D)* *+ tet*(E) *+ dfr12 + sul1 + bla_TEM1_*
08045	*A. hydrophila*	silver hatchet	Guyana	Amx,Otc,Tet,Eno,Cpr,Gat,Ofl,Flq,Oxa, Faz,Fox,Tio,Chl, Tob(I),Stz,Sxt	*qnrS2* * *+ tetD* * *+ Intl1 + dfr13 + sul1*
08046	*A. hydrophila*	three lined pencil	Guyana	Amx,Otc,Tet,Cpr,Eno,Gat,Ofl,Flq,Oxa,Oxa,Faz(I),Fox,Tio(I),Chl,Ffl(I),Str,Stz,Sxt	*tet*(D) *+ tet*(E) *+ dfr12 * ***+*** * qnrS +Intl1 +sul1+* incA/C‡ + *orfF* †
08049	*A. hydrophila*	green guppy	Singapore	Amx,Otc,Tet, Cpr,Eno,Gat,Ofl,Tio,Ffl,Str,Cpr,Gen, Pod,Pip,Tans(I),Oxa,Chl,Flq,Fun,Pod,Stz,Sxt,	*qnrS2* * *+ tet*(A)* *+ aadA1 + sul1 +tet*(D) †
08063	*A. punctata*	blue guppy	Singapore	Amx,Eno,Gen,Tio,Neo,Otc,Tet,Sxt,Ffl,Str,Azt(I),Tans,Fur,Cpr,Gat,Gen,Nit,Fox,Pip,Sxt,Pod, Fun,Oxa,Flq,Mox,Chl,Imi,Stz	*aac61b + qnrS2* *
08078	*A. salmonicida*	silver shark	Singapore	Amx,Otc,Tet,Eno,Faz,Flq,Oxa,Fun,Tio,Ffl,(I),Str,Stz,Sxt	*floR* * + *Intl1 + sul1 + tet*(D)
08081	*A. hydrophila*	salico fantail	Singapore	Amx,Tio,Otc,Tet,Tans,Fox,Fun,Oxa,Flq,Stz(I)	*bla_TEM1_* * *+ tet*(D)
08094	b *A. hydrophila*	*Paracheridon exelrodi*	Colombia	Amx,Eno,Cpr,Gat(I),Tio,Otc,Tet,Str,Fox,Sxt,Stz,Flq,Oxa,Fun,Ofl	*Intl1 + qnrS2 + bla_OXA7_ +sul1 + bla_TEM1_* * *+ tet*(E) *+ dfr12 + aac6lb + aadA1*
08095	*A. hydrophila*	*Corydora melanistus*	Colombia	Amx,Eno,Cpr,Gat,Tio(I),Otc,Tet,Ffl(I),Str, Sxt,Fun,Flq,Ofl,Oxa	*dfr12 + Intl1 + qnrS2 + sul1 + bla_TEM1_* * *+ tet*(D) *+ tet*(E) *+ aadA2 + tet*(C)

Table 1 Footnotes.

a Isolates were identified to provisional species complexes based on partial 16S rRNA gene sequencing and API20NE results.

b Identity also confirmed based on partial *gyrA* and *gyrB* sequencing.

c Abbreviations used: Ami, amikacin; Amx, amoxicillin; Axo, ceftriaxone; Chl, chloramphenicol; Cpr, Ciprofloxacin, Eno, enrofloxacin; Faz, cefazolin; Ffl, florfenicol; Flq, flumequine; Fox, cefoxitin; Fun, furazolidone; Fur, cephalothin; Gat, gatifloxacin; Gen, gentamicin; Imi, imipenem; Mox, moxalactam; Neo, neomycin; Nit, nitrofurantoin; Ofl, ofloxacin; Oxa, oxolinic acid; Pod, cefpodoxime; Str, streptomycin; Pip, piperacillin; Spe, spectinomycin; Str, streptomycin; Sxt, sulphamethoxazole/trimethoprim; Tans, cefotetan Na; Taz, ceftazidime, Tio, ceftiofur; Tob, tobramycin. Codes placed in parentheses indicates isolate was of intermediate tolerance to indicated antimicrobial.

*Indicates gene was identified by both miniaturised microarray and confirmatory PCR.

‡ IncA/C plasmids identified by PCR.

† Indicates gene cassette that was part of a class 1 integron PCR amplified and sequenced from the isolate.

### Antimicrobial Susceptibility Testing

Antimicrobial susceptibility was determined for 94 *Aeromonas* isolates from warmwater species against 34 antibiotics. Methods for disk-diffusion and broth-microdilution assays followed guidelines from the Clinical and Laboratory Standards Institute [24], [25]. Sensititre™ panels (Trek Diagnostic systems, UK) were used for broth microdilution tests, and antibiotic discs (Abtek Biologicals Ltd, Liverpool, UK) for disc-diffusion tests. The MIC values for a subset of isolates to six antimicrobials (Table S4) were also determined using laboratory prepared broth-microdilution assays, as recommended by CLSI [25]. The antimicrobials used in testing were representative of those commonly used to control diseases caused by Gram negative bacteria in human and veterinary medicine, including those used in aquaculture. A total of 33 isolates recovered by Cefas between 1992–2004 from coldwater species, goldfish (*Carassius auratus*; 12/33 tested isolates), koi carp (*Cyprinus carpio*; 15/33 tested isolates) and other species (6/33), were also included in the analysis. These species are typically reared at temperatures less then 20°C. All historical *Aeromonas* isolates were recovered aseptically from the Cefas Bacterial Culture Collection (BCC) held at −80°C in Protect^TM^ vials, freeze dried cultures or in liquid nitrogen.

In the absence of published resistance breakpoints for Aeromonads, tolerance to the antimicrobials tested was determined by examination of the frequency distribution of minimum inhibitory concentration (MIC) and disc diffusion diameter values for all the 127 isolates examined. The boundaries of the populations of isolates showing clearly increased tolerance (non wild type phenotype) and those with higher susceptibility were then defined, with those in between determined as of intermediate (I) susceptibility. Two control strains, *E. coli* ATCC 25922, recommended by both CLSI guidelines, and *A. hydrophila* NCIMB 9240^T^, were also included in parallel in all testing. The range of concentrations of antibiotics and the epidemiological cut-off tolerance values used for both MIC and disc diffusion testing are shown in Tables S1 and S2. All tests were performed at 22±2°C and results read after between 44–48 h incubation.

### Detection of Antibiotic Resistance Genes Using the Identibac AMR-ve™ Miniaturised Micro-Array

A total of 23 isolates were analysed for the presence of 54 different antimicrobial resistance genes using the Identibac AMR-ve™ miniaturised micro-array (http://www.identibac.com/identibac_amr.php). Isolates were analysed following manufacturers instructions as previously described [26], with minor modifications. Isolates were grown overnight at 22°C on Tryptone Soya Agar. Lysates were prepared by suspending a loopful of culture in 400 µl lysis buffer (0.1M Tris HCl, 0.005% Tween 20, Proteinase K). This was incubated at 65°C for 2 h with regular vortexing, followed by heating to 95°C for 15 min. Approximately two micrograms of resultant genomic DNA released from the cells were linearly amplified using the set of antisense primers provided and simultaneously biotin labeled. Single-stranded labeled amplified products were hybridised to the arrays and a signal intensity value was determined for each spot on the array by calculating the quantitative staining value using IconoClust software (version 2; CLONDIAG). The mean signal value for the three replicate spots per probe was used for analysis with a signal intensity greater than 0.3 considered positive, and a signal intensity lower than 0.1 as negative. Those with an intensity value between 0.1 and 0.3 were considered ambiguous.

### PCR Detection of Antibiotic Resistance Genes, Class 1 Integrons and Plasmid Markers

Water bacterial community DNA samples and isolates were also directly screened for a range of resistance genes, class 1 integrons and incompatability group (Inc) A/C and IncN plasmids, using published primers and PCR protocols (Table S3). Template DNA for use in PCR procedures was prepared from isolates by heating colonies suspended in 100 µl molecular grade water at 94°C for 5 min and/or DNAzol™ (Invitrogen) based DNA extraction, following the manufacturer's instructions. The presence of *floR*, IncN and IncA/C markers were initially assessed by multiplex PCR utilizing the HotStarTaq Plus Master Mix Kit (Qiagen) and the primer sets listed in Table S3. PCR conditions were as follows: 5 min activation step at 95°C followed by 35 cycles of 94°C for 1 min, 55°C for 1 min, 72°C for 1 min, and then a final 10 min extension at 72°C.

### General PCR-Conditions and DNA Sequencing

PCR reaction mixtures (50 µl) generally contained sterile molecular-grade water, 1x reaction buffer, 1.5 mM magnesium chloride, 1.25 U (0.25 µl) Go Taq polymerase (Promega, UK), 0.25 mM deoxyribonucleotide triphosphate (dNTPs), and 50 pmol of each primer. 2.5 µl of template was then added to the reaction mixture and samples heated at 94° C for 5 min in a PTC-225 Peltier thermocycler (MJ Research Inc., Massachusetts, USA.). Cycling consisted of 35 cycles of 94°C for 1 min, annealing temperature as indicated for 1 min, 72°C for 1 min, and then a final 10 min extension at 72°C. All isolates positive for the presence of *bla*__TEM_, *florR*, *qnrS*, t*et*(A), *tet*(E), *tet*(D) genes and IncA/C plasmids were reconfirmed by repeat amplication, in parallel with previously negative isolates. For isolates positive for *floR* and IncA/C plasmid markers in the triplex PCR, this was confirmed using separate single target PCR. The identity of a number of the resistance genes was also determined by sequencing the resultant PCR amplicons. In some cases (for class 1 integrons obtained from the isolates and amplicons generated using *qnrS* primers; Table S3), PCR products were cloned using the Promega pGEM-T system (Promega, UK). Sequencing was performed either at the Cefas Weymouth Laboratory, using an ABI 3700 DNA analyser, or by GATC Biotech Germany (see below).

Sequence data was assembled and initially analysed using the Sequencher program (Gene Codes Corp., Ann Arbor, MI, USA).

### Culture-Independent Cloning of Partial Class 1 Integrons from Carriage Water Microbial Communities

For this, approximately 300 ml of each carriage water sample was vacuum filtered through 0.45 µm (Difco) membranes until saturated (3–5 filters per sample). Filters were then placed in 25 ml of molecular grade water (VWR, Leics, UK) in 50 ml falcon tubes (Alpha laboratories, UK). These were then vortexed to resuspend the bacteria. The filters were removed with sterile forceps and the suspension was centrifuged at 3000 g for 15 min. The supernatant was discarded and the pellet was resuspended in 200 µl of molecular grade water by vortexing thoroughly. Template DNA was then extracted using DNAzol™ (Invitrogen) following the manufacturer's instructions. Extractions were stored at −20°C. Partial copies of class 1 integrons were PCR-amplified from the carriage water metagenomic DNA samples using the primers 5CS/3CS (Table S3).

PCR amplicons were cloned as described above. Resultant clones were screened for the presence of inserts by PCR using M13F and M13R primers. Clones that contained inserts were cryopreserved in 50% glycerol. Colonies were then lifted with a sterile wooden pick, and stabbed into ampicillin-supplemented lauria agar wells on a 96 well plate. The plate was then incubated overnight at 37°C prior to transfer to GATC Biotech (Germany) for plasmid extraction and sequencing using M13F and M13R primers.

### Accession Numbers

The DNA sequences of a number of the class 1 integrons, *tet*(A), *tet*(D), *tet*(E) genes and partial 16S rRNA genes obtained in this study were deposited in EMBL under the following accession numbers: FM957852–FM957887.

## Results

### Antimicrobial Susceptibilities

**Figure 1:**
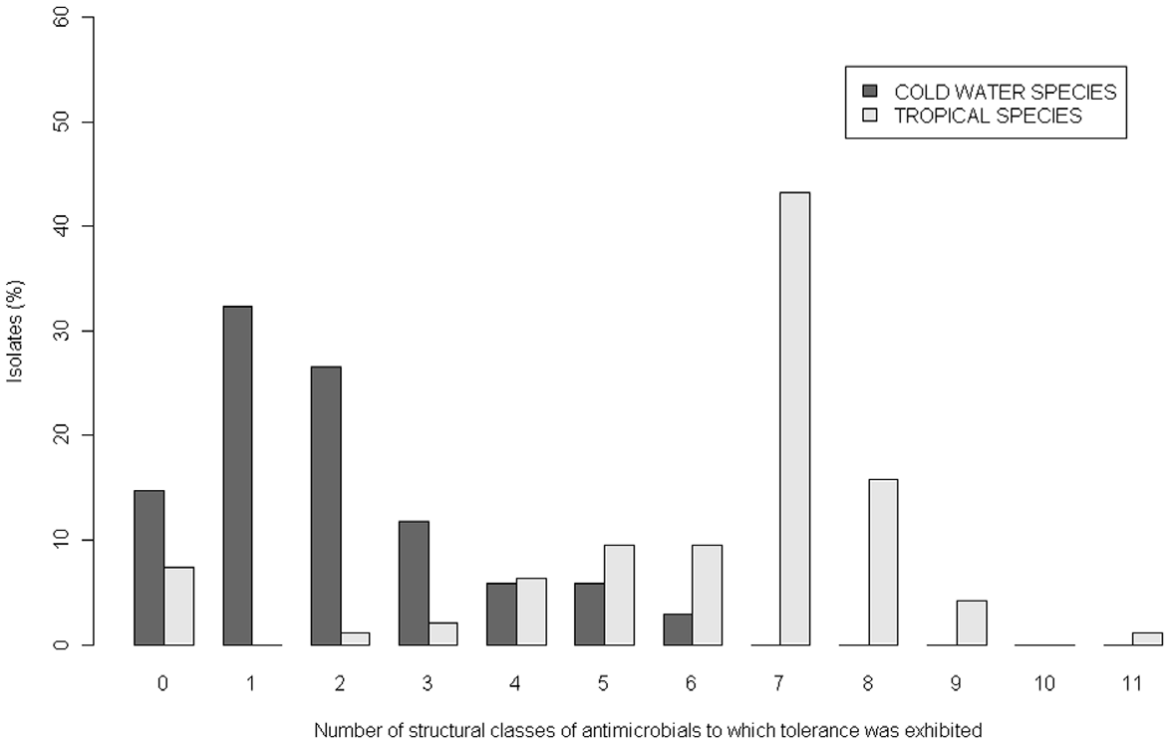
Proportions (%) of isolates recovered from warm water and coldwater species showing tolerance to numbers of different structural classes of antimicrobial. Resistance was seen to representatives of the following eleven structural classes: aminoglycocides, second, third and fourth generation cephalosporins, carbapenems, foliate pathway inhibitors, nitrofurans, phenicols, quinolones, fluoroquinolones and tetracyclines. Note, all isolates also displayed expected wild type resistance to penicillins/first generation cephalosporins (not included in figure).

Half (47/94) of the isolates recovered from warmwater species in 2008 were individually tolerant to ≥15 different antibiotics (Table 2). This multi-drug tolerance (MDT) was broad ranging, with 64% of the isolates shown to be individually tolerant to antimicrobials from seven or more different structural classes of antimicrobial (Figure 1). Many of the isolates recovered from coldwater species, were also shown to be MDT, with 27% individually tolerant to antimicrobials from ≥3 structural classes (Figure 1). There were some antimicrobials that most bacteria tested were highly susceptible to; these included third and fourth generation cephalosporins (ceftriaxone, ceftazidime, cefpodoxime, cefepime and moxalactam) and the carbapenems, imipenem and meropenem (Table 2). However, some isolates were tolerant to these antimicrobials, including an *A. punctata*-like isolate recovered from a Singapore guppy sample (Table 1; isolate 08063). This organism was tolerant to 28 of the antimicrobials tested, including moxalactam, piperacillin, cefpodixime and imipenem (Table 1). The organism was also determined to have heightened tolerance to aztreonam (MIC 16 mg l^−1^) and cefepime (MIC 8 mg l^−1^). The MIC values for six of the antimicrobials were also determined for 27 isolates, including all those listed in Table 1 (Table S4). Isolates were shown to grow in concentrations of up to 384 mg L^−1^ ciprofloxacin (3/27 isolates) and oxytetracycline (14/27 isolates). A number of isolates grew in 768 mg L^−1^ of nalidixic acid (15/27 isolates) and oxolinic acid (7/27 isolates). Isolate 08063 also grew in the highest concentrations tested (768 mgL^−1^) for chloramphenicol and streptomycin (1024 mg L^−1^). A total of 16/27 isolates grew in the highest concentration of suplhadiazine/trimethoprim tested (>730/38.4 mgL^−1^). In total 11/27 of the isolates had MIC values for chloramphenicol of at least 96 mgL^−1^.

**Table 2:** Proportions (%) of warm water and historical cold water species isolates showing atolerance to 34 different antimicrobials.

Antimicrobial	% tolerant isolates (% intermediate tolerant)		Antimicrobial	% tolerant isolates (% intermediate tolerant)	
	cold water species (n = 33)	warm water species (n = 94)		cold water species (n = 33)	warm water species (n = 94)
Oxytetracycline	36	91 (1.1)	Furazolidone	12(12)	66(6)
Tetracycline	24 (18)	85 (6.4)	Amoxicillin	100	100
Flumequine	33 (3.3)	85 (5.3)	Cefazolin	52 (9.1)	83 (11.7)
Oxolinic acid	27 (3)	84 (1.1)	Cefoxitin	12 (12)	52 (4.3)
Enrofloxacin	9.1	77	Cephalothin	3 (6.1)	3.2 (4.3)
Ciprofloxacin	3	63	Cefotetan Na	0	6.3 (1.1)
Ofloxacin	(3.3)	55 (6.4)	Ceftiofur	24 (24)	40 (42.6)
Gatifloxacin	(2.9)	56 (2.1)	Ceftazidime	0	0
Chloramphenicol	6	56 (3)	Ceftriaxone	0	(1.1)
Florfenicol	0	19.1 (17)	Moxalactam	0	5.3
Amikacin	6.1	5.3	Cefpodoxime	0	4.3 (1.1)
Gentamicin	6.1	31	Piperacillin	3	7.4 (10.5)
Neomycin	6.1	39 (10.6)	Cefepime	0	0 (1.1)
Spectinomycin	33	77	Aztreonam	6	(1.1)
Streptomycin	6.1 (9.1)	49 (20)	Imipenam	0	2.1
Tobramycin	3 (3)	61 (12.8)	Meropenem	0	0
bSXT	6.1	67 (1.1)	Nitrofurantoin	0	1.1 (3.2)

94 warm water species isolates and 33 coldwater species isolates were tested in total.

a Testing was done in compliance with CLSI guidelines (CLSI 2004a; CLSI 2004b). Range of concentrations of antimicrobials tested and interpretative tolerance criteria used shown in Tables S1-2.

b SXT  =  sulphamethoxazole/trimethoprim.

### Miniaturised Microarray and PCR Detection of Antibiotic Resistance Genes, Class 1 Integrons and Plasmid Markers in *Aeromonas* spp. Isolates

A total of 23 isolates were analysed for the presence of 54 different antimicrobial resistance genes using the Identibac AMR-ve™ miniaturised micro-array. DNA probes for a range of different resistance genes hybridized with DNA prepared from the *Aeromonas* isolates (Table 1). Positive probes included those directed at genes mediating resistance to tetracyclines, with samples positive by this method for the presence of *tet*(A), *tet*(C), *tet*(D), *tet*(E) and *tet*(G). The isolates were also tested in parallel using conventional PCR-based detection for *tet* A-F (Table S3). The presence of *tet*(A), *tet*(D) and *tet*(E) was also confirmed by both PCR and DNA sequencing in a number of isolates. However there were some discrepancies, with a number of isolates positive for the presence of *tet* genes by miniaturised microarray, even though these genes could not be detected by PCR (Table 1). These isolates were also nearly all tolerant to tetracycline and oxytetracycline (Tables 1 and S4).

Genes mediating resistance to betalactams (*bla*__OXA7_ and *bla*__TEM1_) were detected in many of the isolates by microarray and, in the case of *bla*__TEM1_, PCR. A number of isolates were also positive for the presence of *qnrS* using the miniaturized miocroarray. All these isolates and a number of other isolates were also tested in parallel by PCR for *qnrS* by PCR, with 47 of 94 recently isolated bacteria from warmwater species, shown to be positive by this alternative method. One organism originally isolated in 1998 (isolate 98013; Table 1) was also positive. Out of these 48 amplicons, 24 were double-digested with the restriction enzymes *Hha*I and *Rsa*I and shown to share the same restriction profile. Four amplicons were sequenced and shown to share 100% identity with a *qnrS2* sequence (EU439941), derived from an *Aeromonas* sp. isolated from the river Seine in France [27].

The florfenicol and chloramphenicol resistance gene *floR* [28] was detected by miniaturized microarray in three out of 23 isolates. Follow up PCR analysis confirmed the presence of this gene in 16 out of 93 recent bacterial isolates from ornamental fish species. It was not detected in any of the historical coldwater isolates. Additionally, the *floR* amplicon was detected in 18/21 of the carriage water microbial community DNA samples (not shown).

Correlating with observed resistance to aminoglycocides, aminoglycoside transferase genes (*aadA1*, *aadA2*, *aac61b*) were detected in isolates.(e.g. isolates 93024, 08041, 08049, 08063, 08094 and 08095; Table 1) Miniaturized microarray analysis also identified the likely presence of dihydrofolate reductase (*dfrA1*, *dfrA12*, *dfrA13*), as well as *sul1*, that mediates resistance to sulmethoprim, in a range of isolates that were resistant to sulphamethoxazole/trimethoprim (Table 1). Fifty percent (56/112) of the isolates tested were also confirmed as positive for class 1 integrases by PCR. Additionally, DNA sequencing of class 1 integron PCR amplicons from five example isolates identified antibiotic resistance gene cassettes (Table 1). These included confirming the presence of genes also detected by microarray in these isolates (*dfrA1* and *dfrA12*, *aadA*1 and *aadA*2), a gene encoding a quaterinary ammonium drug pump *qacE2, arr2* that mediates rafampacin resistance, as well as the expected *int1* gene(Table 1). Two gene cassettes that encode proteins of unknown function, that have also been identified by other workers in class 1 integrons from a variety of clinical bacterial isolates, were also identified, *orf*F and *orf*C. Three of the isolates were also shown to be positive for the IncA/C plasmid marker by PCR, but none of the isolates were positive for IncN plasmid markers (Fig. S1). Plasmid markers were detected by PCR in 8/21 (IncN) and 11/21 (IncA/C) carriage water microbial community DNA samples (not shown).

### Culture Independent Cloning of Partial Class 1 Integrons from Carriage Water Microbial Communities

The class 1 integrons and associated gene cassettes present in the microbial communities in selected water samples were also examined using a culture-independent approach. Copies of partial class 1 integrons were directly PCR-amplified from water samples, cloned into *E. coli* and sequenced. The inserts from a total of 58 clones obtained from five carriage water samples were sequenced and shown to be between 103 and 808 bases in length. Initial BLAST comparisons [29] with GenBank deposited DNA sequences determined that 39 of these inserts contained copies of sections of class 1 integrons. Further comparison with class 1 integrons in the Integrall database (http://integrall.bio.ua.pt/) showed that these partial class 1 integrons contained a number of different gene cassettes, including; dihydrofolate reductase types *dfrA1*, *dfrA17*, *dfrA5*, *dfrA21*, *dfrA22*, *dfrA23*; the aminoglycoside adenyltransferase types *aadA1*, *aadA2*; and the quaternary ammonium resistance drug pump-associated gene *qacE2* (Table 3). The 19 other inserts sequenced contained other cloned sections of microbial community DNA (partial copies of bacterial DNA encoding polymerase genes and other bacterial DNA; data not shown).

**Table 3:** Resistance gene cassettes identified in copies of class 1 integrons obtained by PCR directly from samples of carriage water microbial community DNA and cloned into *E. coli*.

Sample	Origin	Fish species	aNo of sequenced clones containing class 1 integrons	bResistance Gene cassettes identified
1	Guyana	Silver hatchet	9	*aadA1* (2) *aadA2* (2) *dfrA5 dfrA17 dfrA27 qacE2* (4)
2	Manaos	*Corydora reticulata*	7	*qacE2* (4) *afrA1, aadA1* (2)
3	Singapore	Silver Molly	5	*aadA1 aadA2* (2) *dfrA22 dfrA1*
4	Singapore	Silver Shark	7	*DfrA1 aadA1*(3) *aadA2*(1)
5	Colombia	*Corydora arcuatus*	11	*qacE2* (3) *aadA1* (3) *dfrA21 dfrA22* (3) *dfrA23*

Table 3 footnotes.

a Resistance gene cassettes were identified by comparison with sequences in the integrall database http://integrall.bio.ua.pt/. The sequences of nine of the 38 partial class 1 integron DNA sequences obtained were deposited in EMBL under the accession numbers FM957877 to FM957885.

b *aad*, aminoglycoside adenylyltransferase, encoding streptomycin-spectinomycin resistance protein; d*fr*, dihydrofolate reductase genes mediating trimethoprim resistance; *qacE2*, gene encoding a quaternary ammonium resistance compound protein (multidrug pump); intI1, integrase: site specific recombination (*attI* and *attC* site).

## Discussion

Other studies have also reported high levels of resistance in bacteria isolated from warmwater ornamental species [3], [5]. Tolerance to many of these antimicrobials has likely been driven by their use in the pet fish trade. In particular, oxytetracycline, nitrofurans (e.g. furazolidone), potentiated suphonamides, and oxolinic acid, which many of the tested organisms showed high tolerance to (Table 2 and Table S4), have been used for many years [3]; Cefas Fish Health Inspectorate Staff, Personal Observations). Two small-scale surveys in the USA in the early 1990's also showed a difference in relative tolerance between isolates recovered from warmwater and coldwater species [3], [30].

Tolerance to tetracyclines was particularly widespread across all screened isolates (Table 2). It is well established that transferable *tet* genes are widely disseminated in the aquatic fish farming environment [11]–[14], [31], and similar genes were identified in some of the isolates in this study by both PCR and miniaturized microarray. There were also a number of isolates containing DNA that hybridized with *tet* probes in the microarray, but were otherwise negative for these and similar genes by PCR. One explanation could be that these isolates contained novel variants of known *tet* genes that hybrized with the probes used for miniaturized microarray analysis, but which were not complementary to the PCR primer sets used. More detailed genetic characterization, that was beyond the scope of this study (e.g. whole genome sequencing or tetracycline-directed cloning), is likely required to accurately determine the genetic basis to tetracycline resistance in these isolates. It should be borne in mind that the design of the probes on the array was biased toward known sequences of Gram negative organisms of human health significance.

There were also high levels of tolerance observed to all the quinolones and fluoroquinolones, particularly in the organisms isolated from warmwater species (Table 2; Table S4). It is interesting to note that Dixon et al. [3] only reported relatively low tolerance to the fluoroquinolone, sarafloxacin, included in that study. Although care should be taken in directly comparing results from limited surveys generated using different methodologies, it is possible that the tolerance of pet fish associated bacteria to the fluoroquinolones has increased since they were first introduced for widespread use in clinical and veterinary medicine in the 1980s. Such tolerance is often mediated by mutations to chromosomal genes [32], with resistance in a number of aquatic bacterial species linked to changes in the genes coding for DNA gyrase and topoisomerase IV enzymes [22], [33]–[34]. The quinolone resistance determining regions of the *gyrA* and *gyrB* genes of five representative isolates were sequenced (isolates 08020, 08030, 08033, 08043 and 08094; Table 1), with no coding mutations noted, suggesting other mechanisms may be responsible. Transferable, plasmid-mediated resistance is increasingly recognized [35]. The finding of *qnrS*2 [27] at such high prevalence, in historical and more recent *Aeromonas* isolates recovered from ornamental fish suggests it may be ubiquitous in bacteria in the ornamental fish trade. Its role in observed tolerance to quinolones and fluoroquinolones in isolates carrying the gene is equivocal as this gene typically mediates only low level resistance to these antimicrobials.

Chloramphenicol and florfenicol tolerance was also observed for many of the organisms associated with warmwater species (Tables 1 and 2). It was also shown that a relatively high proportion of the isolates were also positive for *floR*. Dissemination of genes conferring resistance to florfenicol is of concern, as in many countries, including the UK, it has only relatively recently been licensed for use in animals, including fish, destined for human consumption. Many of the isolates also contained genes coding for Beta-lactamases, with *bla*_TEM−1_ and *bla*_OXA-7_ both detected (Table 1). Apart from the *A. salmonicida* isolate tested (08078), the *Aeromonas* spp that were found to carry these two genes are typically considered intrinsically resistant to the first generation cephalosporins and narrow spectrum penicillins they mediate resistance to. It is possible that these genes were acquired in association with functionally more useful genes, coded by plasmids or other transferable elements. As some tolerance was also noted to third generation cephalosporins (Tables 1 and 2), it is suggested that this be investigated further to determine if this tolerance was mediated by transferable elements as transfer of these elements could be of clinical significance.

A total of 50% of the isolates were positive for class 1 integrons, similar to the levels reported in a survey of motile Aeromonads recovered from freshwater fish farms [12] and the 35% reported for isolates recovered from a slaughterhouse wastewater treatment plant [36]. The proportions of bacteria that were positive for class 1 integrons appear much lower in the other aquatic environments that have so far been sampled [37]–[39].

Carriage of *dfr*, *sul* and *aad* genes may have contributed to the high levels of resistance noted to both foliate pathway inhibitors and aminoglycocides seen, particularly in the organisms associated with warmwater species (Table 1). Comparisons with sequences in the Integrall database of integron sequences (http://integrall.bio.ua.pt/) showed that very similar arrangements of resistance gene cassettes to those found in bacterial isolates and water microbial communities have previously been described in class 1 integrons found in other human, fish and terrestrial animal pathogens. These included those associated with clinical and environmental A*eromonas* isolates from Taiwan [20].

All 39 of the class 1 integrons identified in the constructed clone library contained gene cassettes that have also been recovered from human and veterinary clinical isolates. All but one of the 40 gene cassettes characterized was associated with antibiotic and biocide resistance. This suggests that the carriage water microbial communities examined were enriched for class 1 integrons containing antimicrobial resistance gene cassettes.

The detection of IncA/C plasmids in three of the isolates recovered from warm water species was noteworthy. Recent work [15]–[17] has shown that IncA/C plasmids are responsible for self-transmissible antibiotic resistance in North American aquaculture pathogens, as well as being increasingly important in veterinary and human medicine [15], [40]–[42]. Preliminary conjugal transfer experiments, using tetracycline resistance for selection, showed successful transfer of IncA/C plasmid markers associated with one of the three positive isolates (isolate 08020; Table 1) to *Yersinia ruckeri* and subsequently to *E. coli* ATCC25922 (using methodology described in [15]). In the context of *Aeromonas* spp. as reservoirs of this clinically important class of plasmids, it is noteworthy that the original IncA/C reference plasmid pRA1 was recovered from a fish-pathogenic *A. hydrophila* isolate in 1971 [42], [43]. Work assessing the importance of plasmid-mediated resistance in the identified tolerant isolates is ongoing.

Isolates were identified here that exhibited tolerance to agents from a number of different structural classes, synthetic (i.e. nalidixic acid, sulphamethoxazole/trimethorprim) as well as naturally derived agents, and to relatively new antimicrobials recently introduced in human medicine (i.e. ciprofloxacin) (Table S4). Of these resistant isolates, many demonstrated resistance to multiple antibiotics in the hundreds of mg per liter range, (Table S40. These observations suggest a ‘superbug’ phenomenon, whereby multi-antibiotic resistant isolates also demonstrate higher overall resistance levels. Enne *et al*. [44] postulate that low fitness costs are associated with multi-antibiotic resistance in *E. coli*. The authors noted that, once established, combinatorial resistances (particularly facilitated via mobile genetic elements such as plasmids) might be difficult to eliminate through reduction in prescribing alone. These results imply that this process may not be restricted to established pathogenic or opportunistic bacteria, but a phenomenon common in environmental bacteria, or bacteria with established environmental reservoirs.

### Conclusions

A surprisingly high level of antimicrobial tolerance was identified in bacteria associated with warmwater ornamental species and ornamental fish carriage water. The significance of these tolerant bacteria from ornamental fish in acting as a potential reservoir for mobilisable antibiotic resistance should be systematically assessed. This should help prevent the potential spread of resistance to pathogens of human and animal health importance, and improve fish welfare and treatment. Antibiotic use for prophylactic purposes should also ideally be replaced by better husbandry and transport conditions and the use of vaccination where available.

## Supporting Information

Figure S1 Multiplex detection of the florfenicol resistance gene, floR, and markers for the IncA/C and IncN plasmids.
(0.46 MB PPT)

Table S1 Tolerance cut off values and range of concentrations of antimicrobials used in broth microdilution testing [24]. Also shown are the ranges in MIC values recorded for the two control strains included in parallel during testing, *E. coli* NCIMB 25922 and *A. hydrophila* NCIMB 9240T
(0.05 MB DOC)

Table S2 Interpretative tolerance cut offs and range of concentrations of discs used for disc diffusion testing [25].
(0.03 MB DOC)

Table S3 Primers and PCR conditions used in study.
(0.03 MB DOC)

Table S4 MIC values (Î¼g/ml) determined for selected isolates to seven antimicrobials.
(0.06 MB DOC)

## References

[1] Wittington RJ, Chong R (2007). Global trade in ornamental fish from an Australian perspective: The case for revised import risk analysis and management strategies.. Prev Vet Med.

[2] OATA website http://www.ornamentalfish.org/aquanautmarket/market.php) (6 October 2009 Last accessed)

[3] Dixon BA, Yamashita J, Evelyn F (1990). Antibiotic resistance of *Aeromonas* spp. isolated from tropical fish imported from Singapore.. J Aquat Anim Health.

[4] Cole B, Tamaru CS, Bailey R (1999). Shipping practices in the Ornamental Fish Industry. Center for Tropical and Subtropical Aquaculture, Publication No. 131.

[5] Trust, TJ, Whitby, JL (1976). Antibiotic resistance of bacteria in water containing ornamental fishes.. Antimicrob Agent Chemother.

[6] D'Costa V, McGrann KM, Hughes DW, Wright GD (2006). Sampling the antibiotic resistome.. Science.

[7] Levy, SB, Marshall B (2004). Antibacterial resistance worldwide: causes, challenges and responses.. Nature Med Rev.

[8] O'Brien TF (2002). Emergence, spread and environmental effect of antimicrobial resistance: how use of an antimicrobial anywhere can increase resistance to any antimicrobial anywhere else.. Clin Infect Dis.

[9] Singer R, Ward MP, Maldonado G (2006). Can landscape ecology untangle the complexity of antibiotic resistance? Nature Rev.. Microbiol.

[10] Hall RM, Collis CM, Kim M J, Partridge SR, Recchia GD (1999). Mobile gene cassettes and integrons in evolution.. Ann N Y Acad Sci.

[11] Rhodes G, Huys G, Swings J, McGann P, Hiney M (2000). Distribution of oxytetracycline resistance plasmids between Aeromonads in hospital and aquaculture environments: implication of Tn1721 in dissemination of the tetracycline resistance determinant *tetA*.. Appl Environ Microbiol.

[12] Schmidt AS, Bruun MS, Dalsgaard I, Larsen JL (2001). Incidence, distribution and spread of tetracycline resistance determinants and integron- associated antibiotic resistance genes among motile Aeromonads from a fish-farming environment.. Appl Environ Microbiol.

[13] Furushita M, Shiba T, Maeda T, Yahata M, Kaneoka A (2003). Similarity of tetracycline resistance genes isolated from fish farm bacteria to those from clinical isolates.. Appl Environ Microbiol.

[14] Sørum H, L'Abée-Lund TM, Solberg A, Wold A (2003). Integron-containing IncU plasmids pRAS1 and pAr-32 from the fish pathogen *Aeromonas salmonicida*.. Antimicrob Agents Chemother.

[15] Welch TJ, Fricke WF, McDermott PF, White DG, Rosso M-L (2007). Multiple Antimicrobial Resistance in Plague: An Emerging Public Health Risk.. PLoS ONE.

[16] Welch TJ, Evenhuis J, White DG, Patrick F, McDermott PF (2008). IncA/C plasmid-mediated florfenicol resistance in the catfish pathogen *Edwardsiella ictaluri*.. Antimicrob Agent Chemother.

[17] McIntosh D, Cunningham M, Ji B, Fekete FA, Parry EM (2008). Transferable, multiple antibiotic and mercury resistance in Atlantic Canadian isolates of *Aeromonas salmonicida* subsp. *salmonicida* is associated with carriage of an IncA/C plasmid similar to *the Salmonella enterica* plasmid pSN254.. J Antimicrob Chemother.

[18] Musto J, Kirk M, Lightfoot D, Combs, Mwari L (2006). Multi drug resistant Salmonella Java infections acquired from tropical ornamental fish aquarium, Australia, 2003-04.. Commun Diseas Intell.

[19] Anon (1999). Report on Microbial Resistance in Relation to Food Safety. Advisory Committee on the Microbiological Safety of Food..

[20] Chang Y, Shih DY, Wang J, Yang S (2007). Molecular characterization of class 1 integrons and antimicrobial resistance in *Aeromonas* strains from foodborne outbreak-suspect samples and environmental sources in Taiwan.. Diagn Micr Infec Dis.

[21] Pond MJ, Stone DM, Alderman DJ (2006). Comparison of conventional and molecular techniques to investigate the intestinal microflora of rainbow trout Oncorhynchus mykiss).. Aquaculture.

[22] Goñi-Urriza M, Arpin C, Capdepuy M, Dubois V, Caumette P (2002). Type II topoisomerase quinolone resistance-determining regions of *Aeromonas caviae*, *A. hydrophila*, and *A. sobria* complexes and mutations associated with quinolone resistance,. Antimicrob Agent Chemother.

[23] Rossolini GM, Walsh T, Amicosante G (1996). The *Aeromonas* metallo-beta-lactamases: genetics, enzymology, and contribution to drug resistance.. Microb Drug Resist.

[24] Clinical and Laboratory Standards Institute (2004a). Methods for Broth dilution Susceptibility Testing of Bacteria Isolated from Aquatic Animals; Proposed Guideline, M49*-P.* CLSI, Wayne, PA, USA..

[25] Clinical and Laboratory Standards Institute (2004b). Methods for Antimicrobial Disk Susceptibility Testing of Bacteria Isolated from Aquatic Animals; Proposed Guideline, M42-*P.* CLSI, Wayne, PA, USA..

[26] Batchelor R, Hopkins K L, Liebana E, Slickers P, Ehricht R (2008). Development of a miniaturised micro-array for the rapid identification of antimicrobial resistance genes in Gram-negative bacteria.. Int J Antimicrob Ag.

[27] Cattoir V, Poirel L, Aubert C, Soussy C-J, Nordmann P (2008). Unexpected occurrence of plasmid-mediated quinolone resistance determinants in environmental *Aeromonas* spp.. Emerg Infect Dis.

[28] Smith P (2008). Aquaculture and florfenicol resistance in *Salmonella enterica Typhimurium* Dt104.. Emerg Infect Dis.

[29] Altschul SF, MaddenTL, Schäffer AA, Zhang J, Zhang Z (1997). Gapped BLAST and PSI-BLAST: a new generation of protein database search programs.. Nucleic Acids Res.

[30] Dixon BA, Issvoran G (1993). Antibacterial drug resistance in *Aeromonas* spp. isolated from domestic goldfish and koi from California.. J. World Aqua Society.

[31] Jacobs L, Chenia HY (2007). Characterization of integrons and tetracycline resistance determinants in *Aeromonas* spp. isolated from South African aquaculture systems.. Int J Food Microb.

[32] Ruiz J (2003). Mechanisms of resistance to quinolones: target alterations, decreased accumulation and DNA gyrase protection.. J Antimicrob Chemother.

[33] Giraud E, Blanc G, Bouju-Albert A, Weill F-X, Donnay-Moreno C (2004). Mechanisms of quinolone resistance and clonal relationship among *Aeromonas salmonicida* strains isolated from reared fish with furunculosis.. J Med Microbiol.

[34] Izumi S, Aranishi F (2004). Relationship between gyrA mutations and quinolone resistance in *Flavobacterium psychrophilum* isolates.. Appl Environ Microbiol.

[35] Robicsek A, Jacoby GA, Hooper DC (2006). The worldwide emergence of plasmid-mediated quinolone resistance. Lancet Infect Dis..

[36] Moura A, Henriques I, Ribeiro R, Correia A (2007). Prevalence and characterization of integrons from bacteria isolated from a slaughterhouse wastewater treatment plant.. J Antimicrob Chemother.

[37] Rosser SJ, Young HK (1999). Identification and characterisation of class 1 integrons in bacteria from an aquatic environment.. J Antimicrob Chemother.

[38] Gaze, WH, Abdouslam N, Hawkey PM, Wellington EMH (2005). Incidence of class 1 integrons in a quaternary ammonium compound-polluted environment.. Antimicrob Agents Chemother.

[39] Gillings MR, Krishnan S, Worden PJ, Hardwick SA (2008). Recovery of diverse genes for class 1 integron-integrases from environmental DNA samples.. FEMS Microbiol Lett.

[40] Egorova S, Timinouni M, Demartin M, Granier SA, Whichard JM (2008). Ceftriaxone- resistant Salmonella enterica Serotype Newport, France.. Emerging Infectious Diseases.

[41] Pan JC, Ye R, Wang HQ, Xiang HQ, Zhang W (2008). *Vibrio cholerae* O139 multiple drug resistance mediated by *Yersinia pestis* pIP1202-like conjugative plasmids.. Antimicrob Agents Chemother.

[42] Fricke WF, Welch TJ, McDermott PF, Mammel MK, LeClerc JE (2009). Comparative genomics of the IncA/C multidrug resistance plasmid family.. J Bacteriol.

[43] Aoki T, Egusa S, Ogata Y, Watanabe T (1971). Detection of resistance factors in fish pathogen *Aeromonas liquefaciens*.. J Gen Microbiol.

[44] Enne VI, Livermore DM, Stephens P, Hall LM (2001). Persistence of sulphonamide resistance in *Escherichia coli* in the UK despite national prescribing restriction.. Lancet.

