# Supplementary material for: High Prevalence of Multidrug-Tolerant Bacteria and Associated Antimicrobial Resistance Genes Isolated from Ornamental Fish and Their Carriage Water
Source: PLoS One. 2009 Dec 21;4(12):e8388. doi: 10.1371/journal.pone.0008388 (PMC2793012; doi:10.1371/journal.pone.0008388)
Supplement: Figure S1 — Multiplex detection of the florfenicol resistance gene, floR, and markers for the IncA/C and IncN plasmids. (0.46 MB PPT) [file pone.0008388.s001.ppt]

## Slide 1
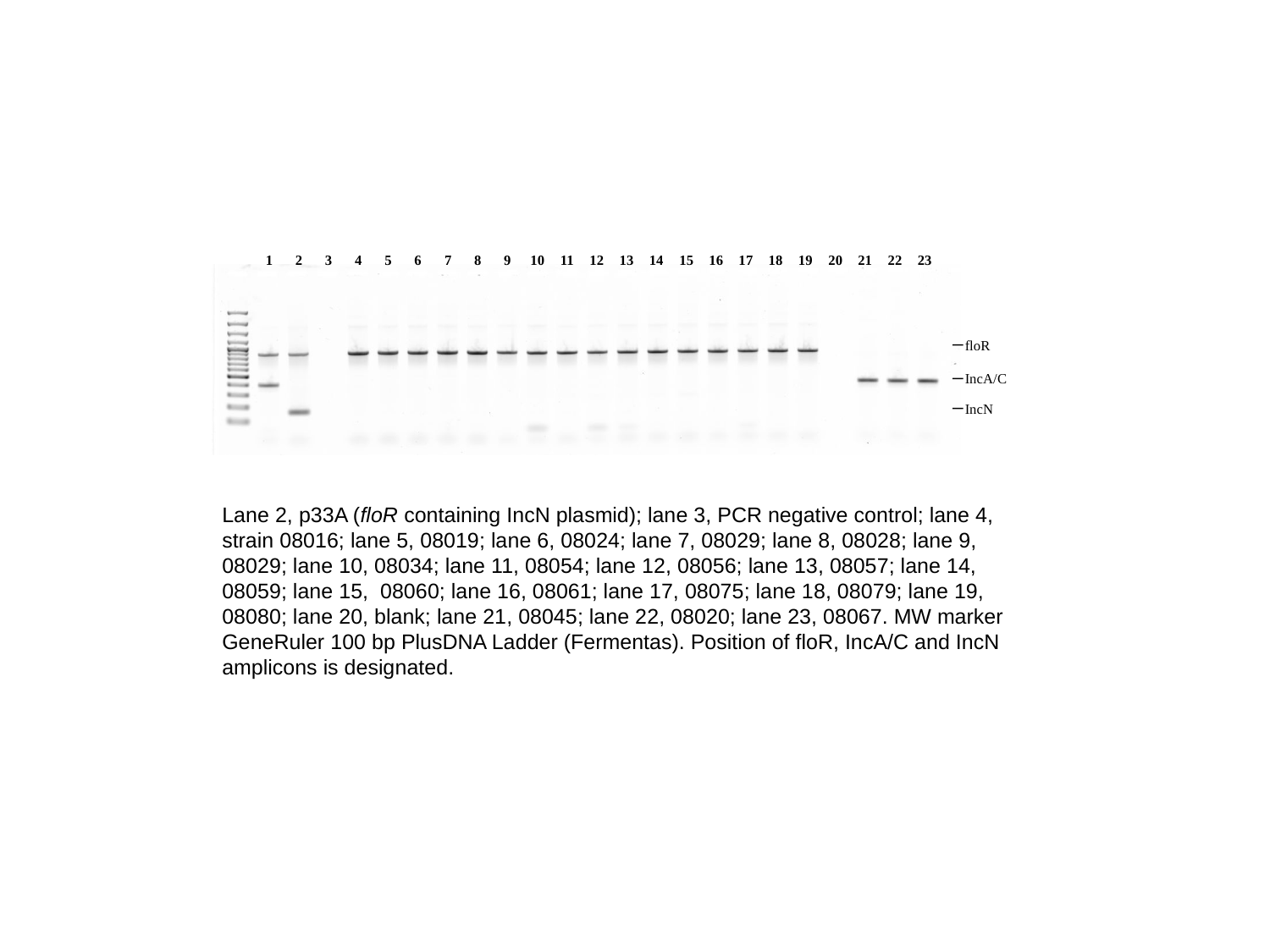

1
2
3
4
5
6
7
8
9
10
11
12
13
14
15
16
17
18
19
20
21
22
23
floR
IncA/C
Lane 2, p33A (floR containing IncN plasmid); lane 3, PCR negative control; lane 4, strain 08016; lane 5, 08019; lane 6, 08024; lane 7, 08029; lane 8, 08028; lane 9, 08029; lane 10, 08034; lane 11, 08054; lane 12, 08056; lane 13, 08057; lane 14, 08059; lane 15, 08060; lane 16, 08061; lane 17, 08075; lane 18, 08079; lane 19, 08080; lane 20, blank; lane 21, 08045; lane 22, 08020; lane 23, 08067. MW marker GeneRuler 100 bp PlusDNA Ladder (Fermentas). Position of floR, IncA/C and IncN amplicons is designated.
IncN
