# Supplementary material for: High Prevalence of Multidrug-Tolerant Bacteria and Associated Antimicrobial Resistance Genes Isolated from Ornamental Fish and Their Carriage Water
Source: PLoS One. 2009 Dec 21;4(12):e8388. doi: 10.1371/journal.pone.0008388 (PMC2793012; doi:10.1371/journal.pone.0008388)
Supplement: Table S1 — Tolerance cut off values and range of concentrations of antimicrobials used in broth microdilution testing [24]. Also shown are the ranges in MIC values recorded for the two control strains included in parallel during testing, E. coli NCIMB 25922 and A. hydrophila NCIMB 9240T (0.05 MB DOC) [file pone.0008388.s002.doc]

| Antimicrobial | Range tested (μg/ml) | Interpretation of MIC tolerance (T) values | | Range in values for Control strains | | Antimicrobial | Range tested (μg/ml) | Interpretation of MIC values | | Range in values for Control strains | | |
| --- | --- | --- | --- | --- | --- | --- | --- | --- | --- | --- | --- | --- |
|  |  | T | S≤ |  |  |  |  | T | S≤ | ATCC  25922 | NCIMB  9240^T^ |  |
| Amikacin | 8-64 | 32 | 8 | <8 | <8 | Gatifloxacin | 1-8 | 8 | 2 | <1 | <1 | |
| Amoxicillin | 0.25-16 | 16 | 8 | 2-4 | >16 | Gentamicin | 0.5-16 | 8 | 4 | <0.5 | <0.5 | |
| Aztreonam | 8-32 | 32 | 8 | <8 | <8 | Imipenem | 2-16 | 16 | 4 | <2 | <2 | |
| Cefazolin | 4-32 | 32 | 8 | <4 | >32 | Meropenem | 1-8 | 16 | 4 | <1 | <1 | |
| Cefepime | 4-32 | 32 | 8 | <4 | <4 | Neomycin | 2-32 | 32 | 8 | >32 | <2-2 | |
| Cefotetan Na | 8-32 | 64 | 8 | <8 | <8 | Nitrofurantoin | 16-128 | 128 | 32 | <16 | <16 | |
| Cefoxitin | 4-32 | 32 | 8 | <4-4 | 8-16 | Oxytetracycline | 0.5-8 | 8 | 1 | 0.25-1 | <0.5 | |
| Cefpodoxime | 2-16 | 8 | 2 | <4 | <2 | Piperacillin | 16-128 | 128 | 16 | <16 | <16 | |
| Ceftazidime | 1-32 | 32 | 8 | <1 | <2 | Spectinomycin | 8-64 | 64 | 32 | <8-8 | 16-32 | |
| Ceftiofur | 0.25-4 | 4 | 1 | 0.25-0.5 | 0.5-2 | Streptomycin | 8-1024 | 64 | 8 | <8 | <8-8 | |
| Ceftriaxone | 1-64 | 64 | 8 | <1-1 | <1 | Tetracycline | 0.25-8 | 8 | 1 | 0.25-0.5 | <0.25 | |
| Cephalothin | 4-32 | 32 | 8 | <4-4 | <4-8 | ^a^SXT | 0.5/9.5-4/76 | 8/152 | 2/38 | <0.5/9.5 | <0.5/9.5 | |
| Ciprofloxacin | 0.5-4 | 4 | 1 | <0.5 | <0.5 | Tobramycin | 4-8 | 8 | 4 | <4 | <4 | |
| Enrofloxacin | 0.25-2 | 2 | 0.5 | <0.25 | <0.25 |  |  |  |  |  |  | |
| Florfenicol | 1-8 | 8 | 1 | 2 | <1 |  |  |  |  |  |  | |

^a^SXT = sulphamethoxazole/trimethoprim
