# Supplementary material for: High Prevalence of Multidrug-Tolerant Bacteria and Associated Antimicrobial Resistance Genes Isolated from Ornamental Fish and Their Carriage Water
Source: PLoS One. 2009 Dec 21;4(12):e8388. doi: 10.1371/journal.pone.0008388 (PMC2793012; doi:10.1371/journal.pone.0008388)
Supplement: Table S2 — Interpretative tolerance cut offs and range of concentrations of discs used for disc diffusion testing [25]. (0.03 MB DOC) [file pone.0008388.s003.doc]

| Antimicrobial | Disc content  (μg) | Interpretation of tolerance (T) values. Zone size (mm) | | Range in zone sizes (mm) for control strains | |
| --- | --- | --- | --- | --- | --- |
|  |  | T≤ | S | ATCC  25922 | NCIMB  9240^T^ |
| Chloramphenicol | *25 | 10 | 15 | 24-27 | 27-49 |
| Enrofloxacin | 5 | - | - | 38-45 | 36- >50* |
| Florfenicol | 30 | - | - | 25-29 | 36-52 |
| Furazolidone | 20 | 16 | 21 | 27-33 | 24-2 |
| Flumequine | *4 | 21 | 23 | 32-35 | 35->50* |
| Gentamycin | 10 | - | - | 22-27 | 22-34 |
| Moxalactam | 30 | 19 | 21 | 27-33 | 39->50* |
| Ofloxacin | 10 | 11 | 19 | 40-46 | 36-44 |
| Oxolinic acid | *4 | 13 | 15 | 34-45 | 34-51 |
| SXT | 1.25/23.5 | - | - | 31-34 | 25-32 |
| Tetracycline | 30 | - | - |  |  |

*Disc contents for oxolinic acid, flumequine and chloramphenicol different to those recommended in [25].
