# Supplementary material for: High Prevalence of Multidrug-Tolerant Bacteria and Associated Antimicrobial Resistance Genes Isolated from Ornamental Fish and Their Carriage Water
Source: PLoS One. 2009 Dec 21;4(12):e8388. doi: 10.1371/journal.pone.0008388 (PMC2793012; doi:10.1371/journal.pone.0008388)
Supplement: Table S3 — Primers and PCR conditions used in study. (0.03 MB DOC) [file pone.0008388.s004.doc]

| **Primer type and target** | **Nucleotide sequence (5’ to 3’)** | **^a^Annealing temperature** | **Product size (bp)** | **Reference** |
| --- | --- | --- | --- | --- |
| Class 1 integron | 5CSF: 5’-GGCATCCAAGCAGCAAGC-3’  3CsR: 5’-AAGCAGACTTGACCTGA-3’ | 42 | Variable | [43] |
| Within Class 1 integron | For: 5’-ACCAACCGAACAGGCTTATG-3’  Rev: 5’-GAGGATGCGAACCACTTCCAT-3’ | 47 | ~280 | [43] |
| 16S rRNA | 8F: 5’-AGAGTTTGATCCTGGCTCAG-3’  536R: 5’-GWATTACCGCGGCKGCTG-3’ | 55 | 528 | [44] |
| *Bla*_TEM | For: 5-TCAACATTTCCGTGTCG-3  Rev: 5-CTGACAGTTACCAATGCTTA-3 | 42 | 860 | [45] |
| *Bla_*OXA | For: 5’-TTCAAGCCAAAGGCACGATAG-3’  Rev: 5’-TTCGAGTTGACTGCCGGGTTG-3’ | 59.5 | 700 | [45] |
| qnrS | For: 5’-GCAAGTTCATTGAACAGGGT-3’  Rev: 5’-TCTAAACCGTCGAGTTCGGCG-3’ | 57 | 428 | [46] |
| floR | For: 5’- GATTTTTGGTCCGCTCTCAGAC-3’  Rev:5’- TTTGAACGCAGAAGTAGAACGC-3’ | 55 |  | [47] |
| *tet*(A) | For: 5’-GTAATTCTGAGCACTGTCGC-3’  Rev: 5’-CTGCCTGGACAACATTGCTT-3’ | 62 | ~950 | [47] |
| *tet*(D*)* | For: 5’-ATTACACTGCTGGACGCGAT-3’  Rev: 5’-CTGATCAGCAGACAGATTGC-3’ | 57 | ~1100 | [47] |
| *tet*(E) | For: 5’-GTGATGATGGCACTGGTCAT-3’  Rev: 5’-CTCTGCTGTACATCGCTCTT-3’ | 62 | ~1180 | [47] |

Table S3 Additional references:

[43] Lévesque C, Piché L, Larose C, Roy PH (1995) PCR mapping of integrons reveals several novel combinations of resistance genes. Antimicrob Agents Ch 39: 185-91.

[44] Schlesinger J, Navon-Venezia S, Chmelnitsky I,Hammer-Münz O, Leavitt A, Gold HS, Schwaber MJ, Carmeli Y (2005) Extended-Spectrum Beta-Lactamases among Enterobacter Isolates Obtained in Tel Aviv, Israel. Antimicrob Agents Ch

45 49:1150–56

[45] Cattoir V, Poirel L, Rotimi V, Soussy CJ, Nordmann P (2007) Multiplex PCR for detection of plasmid-mediated quinolone resistance *qnr* genes in ESBL-producing enterobacterial isolates. J Antimicrob Chem 60: 394-97

[46] White DG, Hudson C, Maurer JJ, Ayers S, Zhao S, Lee MD, Bolton L, Foley T, Sherwood J (2000) Characterization of chloramphenicol and florfenicol resistance in *Escherichia coli* associated with bovine diarrhea. J Clin Microbiol 38:4593-4598.

[47] Guardabassi L, Dijkshoorn L, Olsen JE, Dalsgaard A (2000). Distribution of tetracycline resistance determinants A to E and transfer in vitro of tetracycline resistance in clinical and aquatic *Acinetobacter* strains. J Med Microbiol 49: 929-36.
