# Supplementary material for: High Prevalence of Multidrug-Tolerant Bacteria and Associated Antimicrobial Resistance Genes Isolated from Ornamental Fish and Their Carriage Water
Source: PLoS One. 2009 Dec 21;4(12):e8388. doi: 10.1371/journal.pone.0008388 (PMC2793012; doi:10.1371/journal.pone.0008388)
Supplement: Table S4 — MIC values (Î¼g/ml) determined for selected isolates to seven antimicrobials. (0.06 MB DOC) [file pone.0008388.s005.doc]

| Isolate No. | ^a^Identification | Origin | Country  of origin | Otc | Chl | SXT | Str | Na | Oxa | Cip |
| --- | --- | --- | --- | --- | --- | --- | --- | --- | --- | --- |
| 93022 | *A. veronii* | koi carp | UK | 96 | 0.15 | 0.14/0075 | 16 | 48 | 6 | 0.15 |
| 93024 | *A. hydrophila* | koi carp | UK | 48 | 48 | 64 | 16 | 48 | 12 | 0.15 |
| 94070 | *A. hydrophila* | gold fish | UK | 48 | <0.15 | 0.14/0.0075 | 8 | 48 | 0.15 | <0.15 |
| 94071 | *A. hydrophila* | gold fish | UK | 0.15 | <0.15 | 0.14/0.0075 | <8 | 0.15 | <0.15 | <0.15 |
| 97014 | *A. veronii* | gold fish | UK | 0.15 | <0.15 | 0.14/0.0075 | 8 | <0.15 | <0.15 | <0.15 |
| 94126 | *A. hydrophila* | gold fish | UK | 0.15 | 0.15 | 0.14/0.0075 | 8 | 0.15 | <0.15 | <0.15 |
| 98013 | *A. veronii* | koi carp | UK | 96 | <4 | <4 | <8 | 128 | 8 | 3 |
| 98020 | *a. media* | Not known | UK | 0.15 | 0.15 | 0.14/0.0075 | <8 | <0.15 | <0.15 | <0.15 |
| 08015 | *A. punctata* | guppy | Singapore | 384 | 6 | 2.85/0.15 | <8 | >768 | >768 | 384 |
| 08016 | *A. hydrophila* | guppy | Singapore | 384 | 192 | >730/38.4 | 64 | 384 | >768 | 192 |
| 08019 | *A. punctata* | guppy | Singapore | 384 | 192 | >730/38.4 | 32 | >768 | >768 | 384 |
| 08020 | *A. veronii* | guppy | Singapore | 192 | <0.15 | 0.14/0075 | 8 | >768 | >768 | 384 |
| 08022 | *A. hydrophila* | threadfin rainbow | Singapore | 192 | 96 | >730/38.4 | 64 | >768 | >768 | 96 |
| 08030 | *A. hydrophila* | harlequin rasbora | Singapore | 24 | <0.15 | >730/38.4 | 512 | 48 | 24 | 1.5 |
| 08033 | *A. punctata* | redwag platy | Singapore | 384 | 96 | >730/38.4 | 128 | >768 | 384 | 192 |
| 08038 | *A. hydrophila* | three lined pencil | Guyana | 384 | 192 | >730/38.4 | 64 | >768 | 384 | 192 |
| 08039 | *A. hydrophila* | silver hatchet | Guyana | 384 | 3 | >730/38.4 | 128 | >768 | 384 | 192 |
| 08041 | *A. punctata* | silver hatchet | Guyana | 384 | 3 | >730/38.4 | 64 | >768 | 384 | 192 |
| 08043 | *A. hydrophila* | silver hatchet | Guyana | 384 | 192 | >730/38.4 | 128 | >768 | 384 | 96 |
| 08045 | *A. hydrophila* | silver hatchet | Guyana | 384 | 384 | >730/38.4 | <8 | >768 | 96 | 24 |
| 08046 | *A. hydrophila* | three lined pencil | Guyana | 384 | 192 | >730/38.4 | 64 | >768 | 384 | 96 |
| 08049 | *A. hydrophila* | green guppy | Singapore | 384 | 128 | >730/38.4 | 128 | >768 | >768 | 192 |
| 98063 | *A. punctata* | blue guppy | Singapore | 384 | >768 | >730/38.4 | >1024 | >768 | >768 | 192 |
| 08078 | *A. salmonicida* | silver shark | Singapore | 48 | 48 | >730/38.4 | 64 | 384 | 48 | 1.5 |
| 08081 | *A. hydrophila* | salico fantail | Singapore | 384 | <0.15 | 2.85/0.143 | 8 | 192 | 6 | 0.15 |
| 08094 | *A. hydrophila* | *Paracheridon exelrodi* | Colombia | 192 | 96 | >730/38.4 | 256 | >768 | 48 | 96 |
| 08095 | *A. hydrophila* | *Corydora melanistus* | Colombia | 384 | 192 | >730/38.4 | 64 | >768 | 384 | 192 |

Otc = oxytetracycline, Chl= chloramphenicol, SXT = sulphamethoxazole/trimethoprim, Str = stremptomycin, Na = nalidixic caid, Oxa = oxolinic acid, Cip = ciprofloxacin

MIC values for streptomycin were determined using Sensititre^TM^ panels, the other antimicrobials were tested using laboratory-prepared broth microdilution panels, all in compliance with [25].
